# Improving Conceptual Knowledge of the Italian Writing System in Kindergarten: A Cluster Randomized Trial

**DOI:** 10.3389/fpsyg.2018.01396

**Published:** 2018-08-07

**Authors:** Giuliana Pinto, Lucia Bigozzi, Christian Tarchi, Monica Camilloni

**Affiliations:** Department of Education and Psychology, University of Florence, Florence, Italy

**Keywords:** emergent literacy, conceptual knowledge of the writing system, invented spelling, knowledge of letters, orthographic awareness

## Abstract

This study assessed the efficacy of PASSI (*Promoting the Achievement of Sound-Sign Integration*), an intervention to improve children's conceptual knowledge of the Italian writing system in kindergarten, which is an emergent literacy predictor of reading and spelling acquisition focused on letter-speech sound integration. PASSI implements an embedded-explicit approach in which teachers target specific subskills (reflection on the graphic, symbolic and phonological aspect of written signs) and emphasize children's contextualized interactions with oral and written language. One hundred fifty-nine Italian children participated in this study. Six teachers (and their three respective classes) were randomly assigned to the experimental group, and six teachers were assigned to the control group. All children were tested on the invented spelling of words and numbers, knowledge of the alphabet, orthographic awareness, and drawing twice, before and after the intervention. Children's visual-motor integration skills were also assessed as a control variable. The data were analyzed through the complex samples general linear model (GLM) approach. The results confirmed the efficacy of PASSI in promoting children's conceptual knowledge of the writing system and related emergent literacy skills. Theoretical and educational implications of the results are presented and discussed.

## Introduction

This study assessed the efficacy of an intervention to improve children's conceptual knowledge of the Italian writing system in kindergarten. Past studies have shown that children's early competence in this construct are predictive of future reading fluency scores (Bigozzi et al., 2016b) and reading and spelling disorders (Bigozzi et al., 2016a). Children's conceptual knowledge of the writing system are generally assessed through an invented spelling task, in which children create letter-speech sound integrations that correspond to their level of knowledge of the writing system. This factor integrates phonological awareness with grapho-motor skills (Berninger et al., 2008) and visual attention (Germano et al., 2014).

Past studies have demonstrated that children's early literacy skills can be stimulated through educational programs and interventions (Bus and van Ijzendoorn, 1999; Justice and Pullen, 2003). Emergent literacy interventions can be designed through an embedded approach (i.e., emphasizing children's daily self-initiated, naturalistic, and contextualized interactions with oral and written language), an explicit approach (i.e., structured, sequenced and directed instruction targeting specific skills), or a combination of both (Justice and Kaderavek, 2004). Recently, studies have contributed to the reconsideration of phonological awareness as the main predictor of literacy acquisition and brought to light multicomponent constructs, such as children's emergent conceptual knowledge of the writing system (Ouellette and Sénéchal, 2017; Pinto et al., 2017). This construct can be considered the emergent antecedent of the integration process that characterizes formal spelling.

In this study, we focused on children's conceptual knowledge of the Italian writing system to provide kindergarten teachers with an evidence-based intervention that could facilitate reading and spelling acquisition once the children are in primary school. The intervention fosters children's reflection on the characteristics of different symbolic systems used for graphic representations, namely, the invented spelling of words, invented spelling of numbers, invented reading, and drawing skills. In this study, invented spelling and invented reading are defined as children's early attempts to represent words in print before they can conventionally read and spell words (Ouellette and Sénéchal, 2008).

### Learning to spell in the italian language

The target language in this study was Italian, which is characterized by a few differences from other writing systems, including English, the language on which most research on learning to read and spell is based on. Cross-linguistic studies have suggested the existence of a near-universal pattern of reading and spelling development across alphabetic languages (Furnes and Samuelsson, 2010; Ziegler et al., 2010; Caravolas et al., 2012; Landerl et al., 2013), with some moderating effect by the orthographic consistency on the rate of development and patterns of impairments (Paulesu et al., 2001; Seymour et al., 2003; Furnes and Samuelsson, 2010). The two dominant theories describing cross-script diversity in reading and spelling development are orthographic depth and psycholinguistic grain size theory (Daniels and Share, 2018). Orthographic depth refers to the fact that alphabetic orthographies differ by the transparency of their grapheme-phoneme correspondence. Transparent orthographies are characterized as having nearly a 1:1 correspondence, whereas opaque orthographies are characterized by an equivocal grapheme-phoneme correspondence (Katz and Frost, 1992). By implication, phonological skills should be more strongly associated to reading and spelling development in deep orthographies, rather than in shallow ones (Katz and Frost, 1992; Daniels and Share, 2018). According to the psycholinguistic grain size theory, the consistency of spelling–sound mappings may modulate the importance of phonological skills: Whereas in shallow orthographies readers and spellers can rely on single letters (and their phonemic correspondence), in deep orthographies they need to rely on larger grain, such as rhymes (Ziegler and Goswami, 2005; Daniels and Share, 2018; Diamanti et al., 2018).

Interestingly, the rate of reading and spelling development mirrors the transparency of the writing system (Sprenger-Charolles et al., 2011). Italian, the target language in this study, is characterized by a transparent orthography. More specifically, the regularity is higher in grapheme-phoneme relations (forward regularity) than in phoneme-grapheme relations (backward regularity; Wimmer and Mayringer, 2002). For example, when reading, the grapheme “c” corresponds to the phoneme /k/ if followed by a consonant (including “h”) or by one of the following vowels: “a,” “o,” or “u.” In contrast, the same grapheme corresponds to the phoneme /t∫/ when followed by “i” or “e.” There are no exceptions to this rule. However, when writing, the phoneme /k/ can correspond to two different graphemes, “c” as in /kwko/ (“cuoco,” en. tr. “chef”) or “q” as in /kwì/ (“qui,” en. tr. “here”). In Italian, children generally learn to spell through grapheme-phoneme mapping (sublexical procedure), and shift to recognize known words by sight alone (lexical procedure) in later grades (Notarnicola et al., 2012; Bigozzi et al., 2017). Typically, mastery in the sublexical procedure is achieved at the end of first grade (Notarnicola et al., 2012), reaching a ceiling effect at the end of second grade (Cossu et al., 1995). It should be noted that in Italian the regularity of the orthographic system is higher in reading (forward regularity) than it is in spelling (backwards regularity) (Notarnicola et al., 2012; Bigozzi et al., 2016a). When spelling, some phonemes might correspond to one or more graphemes, and correct spelling can be identified only through the context. Finally, developmental studies showed that spelling plays a fundamental role for both, reading and writing acquisition (Pinto et al., 2015a), bringing further support to the importance of spelling in literacy development in Italian.

### Symbolic systems: spelling words, spelling numbers, and drawing

According to the emergent literacy approach, children's preschool competences and knowledge of the nature and conceptual meaning of a writing system begin early in life and influence the formal learning of conventional literacy processes (Whitehurst and Lonigan, 1998; Lonigan et al., 2013). Drawing, numeracy and literacy are all fundamental components of children's emergent understanding of symbolic systems (Whitehurst and Lonigan, 1998) and have been found to be strong predictors of later achievement in formal literacy processes (Yamagata, 2007; Bigozzi et al., 2016b). Past studies have also identified children's ability to differentiate different symbolic systems in kindergarten (e.g., letter from numbers) as a predictor of improvement in reading achievement through primary school (Spira et al., 2005). These symbolic systems share some characteristics. All these symbolic systems allow the expression of mental representations. Both drawing and writing are systems that leave visible marks, unlike what occurs with speaking or reading (Tolchinsky Landsmann, 2003). Drawing can be defined as a process characterized by certain rules that need to be followed (Goodnow and Levine, 1973; Freeman, 1987): Also drawing is characterized by recurrent graphic patterns, such as lines, dots, and circles. However, these symbolic systems differ in some aspects, and children's knowledge and attitudes toward symbols might be domain specific (Tolchinsky Landsmann and Karmiloff-Smith, 1992). Writing is characterized by more restrictions than drawing. The writing system can be segmented into discrete units (i.e., a word can be segmented into letters), and thus, set of units represent a closed system in which nothing can be added without drastically changing its meaning (Tolchinsky Landsmann and Karmiloff-Smith, 1992). Concerning literacy and numeracy, a first relevant difference is that we use an alphabetic system to spell words, whereas numerals can be represented by digits that, in turn, can be spelled in alphabetic writing. Additionally, repeated symbols cannot represent an example of a correct representation of a word (e.g., ppppp), whereas they can represent an example of a correct representation of a number (e.g., 88888). Prior research has found that the conventional use of numbers appears developmentally earlier and more frequently than the conventional use of letters (Yamagata, 2007), suggesting that an intervention targeting children's early attempts at spelling might have different effects on words vs. numbers.

### The construct of conceptual knowledge of the writing system

Conceptual knowledge of the writing system includes two components. The first component is awareness of the existence of different symbolic systems to represent meanings, for instance, written language, numeric language, and drawing (see previous paragraph). The second component is represented by invented spelling, which is the systematic matching of sounds that are included in words with signs that are not necessarily conventional (Liberman, 1971; Read, 1971; Puranik et al., 2011, 2013; Read and Treiman, 2013). Conventional signs are the actual letters of the alphabet. In contrast, “invented” signs are written productions that although not yet letters, include some of the properties of the writing system. Invented spelling refers to children's spontaneous attempts to represent words in print (Read, 1971; Ouellette and Sénéchal, 2017), and several subskills are involved, all of which need to be addressed by an intervention aiming at improving children's conceptual knowledge of the writing system. Certainly, phonological awareness is involved in invented spelling, as children need to be able to discriminate between the sounds included in a word (Vernon and Ferreiro, 1999; Martins and Silva, 2006). This construct also requires children to reflect their level of knowledge of the writing system and provides them with insight into the structure of their writing system (Treiman, 1998; Read and Treiman, 2013). Overall, invented spelling is a developmental step in which children attempt to merge the phonological and orthographic characteristics of a word (Adams, 1998; Ouellette and Sénéchal, 2008). Visual-motor skills are also involved and allow children to apply and execute their knowledge on the phonological-orthographic connectivity (Pinto and Camilloni, 2012; Read and Treiman, 2013).

Several studies have explored children's emerging conceptual knowledge of the writing system and emphasized similarities and differences across languages. In many countries, before the onset of formal schooling, children learn to identify the shapes of letters and known the name of letters (Treiman et al., 2007b); they show some knowledge about the horizontal orientation of their language (Treiman et al., 2007a, 2015); and they show some understanding about the symbolic nature of writing, and how words symbolize meaning in a different way than pictures do (Treiman et al., 2016). Rather than being an all-in-one acquisition, children's emergent conceptual knowledge of the writing system is progressive. For instance, children learn some letters before other ones (Puranik et al., 2013). In a study conducted with 296 preschool children aged 4–5 years, Puranik et al. (2011) found that print knowledge and letter writing were related to name-writing skills, whereas, print knowledge, alphabet knowledge and name writing were related to letter writing skills. Only letter writing skills significantly contributed to the prediction of spelling skills. Thus, letter writing is an important antecedent of spelling, but this knowledge is supported by several other emergent writing skills, confirming the multi-componential nature of children's conceptual knowledge of the writing system. Conceptual knowledge of the writing system was found to be related to literacy acquisition in both transparent (e.g., Italian, Bigozzi et al., 2016a,b) and opaque languages (e.g., English, Ouellette and Sénéchal, 2008).

Taken together, these results suggest that early instruction in conceptual knowledge of the writing system at the preschool level may be promising to enhance emergent as well as formal literacy skills (Puranik et al., 2011).

### Developing conceptual knowledge of the writing system through intervention

There are several reasons to believe that children's conceptual knowledge of the writing system can be improved through an intervention, the most important being that this construct is context dependent. Before entering primary school, children are surrounded by several symbolic representations of the world (Ferreiro, 1988; Ravid and Tolchinsky Landsmann, 2002), and become increasingly able to discriminate written language from other symbolic systems by comprehending several basic features of written language, such as dimensionality, linearity, directionality, horizontality, and finally, letters as a conventional system of shapes (Levin and Bus, 2003; Treiman et al., 2007a,b, 2015; Puranik et al., 2013). Children develop also a pragmatic competence in written language through exposure to adults' use of writing (e.g., shopping list) and the interaction with them or peers in writing-mediated activities (e.g., story-telling) (Aram and Levin, 2004).

Even though most intervention studies have targeted the subskills included in children's conceptual knowledge of the writing system, a few studies have specifically addressed invented spelling. Silva and Martins (2003) verified the efficacy of an invented spelling intervention to foster the development of 30 Portuguese children's phonological awareness. The intervention aimed at leading the child to think about the rules of spelling and to help them move from pre-phonetic to early phonemic spellings. The intervention proved to be effective, suggesting the possibility of promoting both phonological awareness and the gradual learning of the alphabetic principle. In a follow-up study, the authors (Martins and Silva, 2006) suggested that invented spelling intervention programs could replace, or at least complement, phonological awareness programs to prevent difficulties in learning to read. Ouellette and Sénéchal (2008) identified children's invented spelling levels and trained them at a higher level than their own level. The experimental group outperformed the control group in invented spelling, orthographic awareness and the reading of words. Rieben et al. (2009) compared four different early spelling practices that mimicked teaching activities, namely, invented spelling, copied spelling, invented spelling with feedback on orthography, and a control group. According to their results, invented spelling with feedback on orthography was more effective than invented spelling alone or copied spelling in improving children's orthographic awareness but not in the phonologically oriented tasks.

Levin and Aram (2013) referred to these three works and discussed their limitations. According to these researchers, the first two studies (Martins and Silva, 2006; Ouellette and Sénéchal, 2008) designed developmentally tailored interventions that constrained children's progress in their conceptual knowledge of the writing system. In fact, their results were far from optimal, as also discussed by the authors of the original articles. In contrast, Rieben et al. (2009) tested several types of intervention, and although each of the interventions was effective, children's gains were restricted to the type of feedback received. According to Levin and Aram (2013), the problem was that explanations were provided only for the orthographic aspects of the writing system rather than addressing the integrated alphabetic code underlying spelling and reading. To overcome such limitations, Levin and Aram (2013) compared the effects of two mediation routines on children's gains obtained in invented spelling, as well as other early literacy skills. In the process-product mediation group, the experimenter explained the implicit and explicit processes involved in invented spelling immediately after children's invented spelling performance, whereas in the product mediation group, the experimenter showed the correct spelling of a word after students' invented spelling performance. According to their results, the process-product mediation strategy was more effective in enhancing knowledge of letters, as well as the segmentation, spelling and decoding of words, than the product mediation strategy. This result suggests that the explanation of all steps involved in phoneme-grapheme mapping processing along with the display of the correct spelling contributes to the development of early literacy skills, except for naming letters and word decoding. However, this approach has its limitations, as also suggested by the authors. The intervention was adapted to the phono-orthographic characteristics of Hebrew, and invented spelling interventions in other writing systems have to be adjusted to their characteristics.

### Rationale and research questions

Based on these theoretical premises, we developed PASSI (*Promoting the Achievement of Sound-Sign Integration*), an intervention to improve children's conceptual knowledge of the writing system in kindergarten before the onset of formal schooling. There are several differences between PASSI and prior intervention studies in terms of the target skill, intervention design and activities. PASSI is an intervention that includes the simultaneous integration of the dual code, decoding and coding, in three different symbolic systems (word writing, number writing, and drawing), given the importance of early acquisition of the ability to effectively differentiate different symbolic systems (Spira et al., 2005). Children's metacognitive reflection on written language is triggered by activating both children's coding hypotheses (when inventing spelling) and children's decoding hypotheses (when inventing reading). Each of the three symbolic systems shares the need to rely on some conventional rules to be effective, but the rules change from system to system (Spira et al., 2005; Yamagata, 2007). When drawing, children have to include symbols in their production, which should look like the object that they want to represent. In contrast, words and numbers are conventional and arbitrary signs that represent sounds and meanings without any similarity in form. Words represent several types of meanings (concrete and abstract), whereas numbers represent quantities. To effectively convey meaning, words and numbers must also be represented with a conventional syntax. Signs need to be written (and read) in a specific order to create a relationship among them. The same sign can produce different meanings, depending on the symbolic system in which it is included. A circle can be a tire if we are drawing, an “o” if we are spelling words, or a “0” if we are spelling numbers. The simultaneous activation of these three symbolic systems increases children's conceptual awareness of the differences existing between these systems.

To increase children's conceptual knowledge of the writing system, we adopted an embedded-explicit approach to design the intervention, which not only focused on fostering children's spontaneous engagement with the oral and written language present in their natural environment (Read and Treiman, 2013) but also included more systematic, structured skills activities. We targeted specific subcomponents of the invented spelling construct, namely, the graphic, symbolic and phonological aspects of written signs and the relationships among them. The activities were all aimed at stimulating reflection on and the construction of the written sign rather than at anticipating the formal learning of reading and writing. The choice of creating an embedded-explicit approach influenced the research design implemented in this study, a cluster randomized trial, increasing the ecological validity of the study. Since the intervention was delivered by teachers over 15 weeks, we could not randomly assign students to conditions; we had to randomly assign teachers to conditions. The appropriate statistical method was implemented to adjust for intra-cluster correlations.

This study examined the efficacy of PASSI in children's performances in the invented spelling of words and numbers, orthographic awareness and knowledge of letters in the Italian language, with children's visual-motor integration included as a control variable. We hypothesized that PASSI would be more effective in enhancing the invented spelling of words and numbers, orthographic awareness and knowledge of letters than the control group. To test the domain-specific nature of the intervention, we also verified its efficacy in children's drawing skills, hypothesizing that we would not find any significant improvement, notwithstanding its dependency from school practices (Tarchi and Pinto, 2015). Of notice is that emergent literacy and emergent numeracy follow different developmental paths (Yamagata, 2007), which in turn can influence the beneficial effect of PASSI on children's gains.

## Method

### Participants and setting

One hundred fifty-nine Italian children, all attending two different schools located in a city of Central Italy, participated in this study. All children were born in Italy and spoke Italian as their mother-tongue language. At the time of the study, no participant was diagnosed with a physical or mental disability, was included in a diagnostic process, or identified by the teachers as having special educational needs, thus all participants could be defined as typically-developing. Six classes and 12 teachers (two per class) participated. All classes belonged to the same school district, characterized by a middle-high socio-economic level and teaching practices that followed the national guidelines released by the Ministry of Education. In this study, the target language was Italian, which is characterized by a transparent orthography.

In Italy, kindergartens follow national guidelines set by the Ministry of Education and include activities targeting the development of grapho-motor skills, literacy-related skills, and sensorial skills. Children are generally not exposed to formal teaching of reading and spelling, which occurs in first grade. The participating schools were not following any specific program to empower relevant variables for this study and adhered to the national curriculum. All schools and classes were also comparable in terms of the presence, visibility and accessibility of meaningful material for written language. Interviews with the participating teachers confirmed that the experimental group and the control group classrooms did not differ in emergent literacy instruction.

### Procedure

Teachers were considered eligible to the participate in this study if they were tenured, with more than 5 years of teaching experience. The outcome measures were collected in the same manner for both groups. All tests were individually administered and coded by two trained experimenters who were blind to the treatment condition. The pre-test measures were assessed at the beginning of the school year, in October. In November, the experimental group teachers attended a training on the PASSI intervention, which was also offered to the control group teachers once the study was concluded and all data had been collected. The intervention occurred over 15 weeks from mid-January to the end of April. In May, the post-test measures were assessed.

#### Fidelity of implementation

Fidelity of implementation was verified through multiple procedures (O'Donnell, 2008). Teachers received a specific training, as explained above. All teachers included in the experimental group participated in both meetings. During the meetings, the instructor discussed the intervention theory and determined what it meant to implement the intervention with fidelity (O'Donnell, 2008). For each activity, the instructor specified which critical components and processes were necessary to implement the curriculum intervention with fidelity, and which components could be adapted to the classroom by the teacher. Teachers' knowledge and understanding of PASSI were assessed by the instructor.

After the training, each teacher was supervised by a member of the research team that created PASSI. Teachers were provided with a manual, which included detailed description of each activity, to increase the probability of fidelity of implementation (O'Donnell, 2008). The supervisor monitored the implementation of the program through weekly meetings with the teacher, to identify significant deviations from the intervention. The supervisor also held meetings with the control group teachers as part of the school routine practices, during which the supervisor monitored their activity. No sign of contamination with the experimental group was identified in any of the participating classrooms.

A research assistant was assigned to every classroom to assist as a participant observer to take field notes for 20% of the sessions. The field notes confirmed that PASSI was implemented in every classroom without departures from the instructions.

#### Inclusion criteria

**Figure 1:**
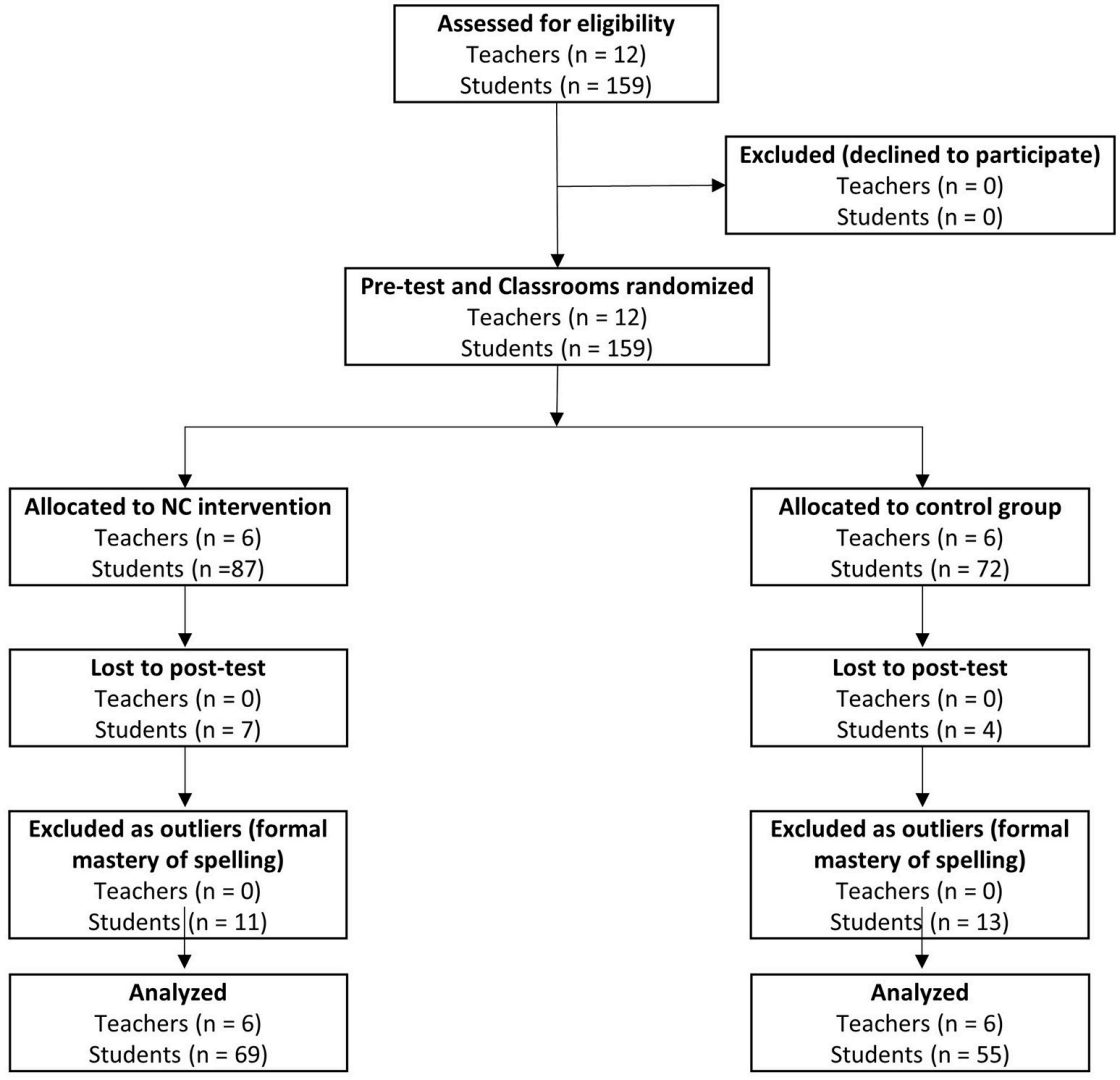
Participants' allocation flow chart.

From the initial sample of 159 children, 11 children (seven children from the treatment group and four children from the control group) were excluded because they did not take part in either the pre-test or post-test assessment or because they were absent at school during the treatment period. As a result, we did not have missing data for the sample used in the analyses. From this sample, we also excluded 24 children (11 children from the treatment group and 13 children from the control group) showing a formal mastery of reading and writing during kindergarten, that is, children who knew how to correctly spell all of the words in the invented spelling of words task and who knew all of the letters of the alphabet as a result of informal extracurricular activities. The final sample included 124 children. The characteristics (number, age, and gender) are described in Table 1. See Figure 1 for a flow chart representing the participants' allocation.

**Table 1:** Sample characteristics (age in months and *n*).

	**Experimental group**				**Control group**			
	***n***	**Age**	**Gender**		***n***	**Age**	**Gender**	
			**M**	**F**			**M**	**F**
3-year-old group	20	45	10	10	16	45	6	10
4-year-old group	23	57	11	12	21	57	14	7
5-year-old group	26	69	7	19	18	69	11	7
Total	69	57	28	41	55	57	31	24

#### Description of experimental groups

##### Experimental group training

PASSI was implemented by the classroom teachers. The six teachers included in the experimental group received a specific training on how to implement the intervention in the classroom. The training consisted of two 2-h meetings with one of the authors of this article. In the first meeting, the researchers explained the theoretical principles of PASSI, whereas the second meeting was a workshop on the actual activities to implement. The researchers explained the activities and given activity sheets. The teachers simulated the activities and received feedback from the instructor. Finally, the teachers' knowledge and understanding of PASSI were assessed by the instructor.

The invented spelling intervention included two aims: (i) to emphasize and enrich the symbolic material present in the child's educational environment (e.g., books, newspapers, magazines, boards, and street signs) and (ii) to create significant contexts in which the symbolic material can be used (e.g., activities on thematic drawing and invented spelling). The activities were designed to be similar to children's everyday routines and to offer the children playful scenarios in which they could concretely use symbolic material. Each activity lasted approximately one and a half hours. Activities were performed twice a week at the beginning of the school day for 3 and a half months (15 weeks). Overall, the children worked on 30 activities, 10 for each category (graphic sign, orthographic sign, and numeric sign). Within each category, five activities stimulated decoding processes, and 5 stimulated coding processes. Each activity involved up to three tasks. At the end of each set of activities, the teacher was encouraged to report his or her observations of the children's contextualized behaviors (e.g., if they are collaborating with peers, frequently requiring the adult, working independently, or working with curiosity) and individual competences (whether the children are completely, partially, or not achieving the activity objectives). The activities varied by type and classroom structure. Some activities were based on activity sheets, some required recycling material, some were games, some others were based on story-telling, and some activities were discussion based. Regarding classroom structure, some activities were addressed to the whole classroom (for instance, discussion-based and game activities), some activities involved small groups, some other activities required students to work in pairs, and some other activities involved individual work. For instance, in the activity “There's mail for you” (targeting the orthographic sign, production), the teacher shows the children several objects involved with mail (e.g., envelopes, stamps, letters, and post-cards). Then, the teacher fosters discussion with the following questions: what is this envelope for? What is a stamp for? What are letters for? Have you ever been to a post-office? What is the difference between a letter and a post-card? Have you ever sent a post-card? What did you want to say with it? The activity concludes with the teacher showing a few examples of letters so that the children familiarize themselves with this type of writing. Finally, the children work in pairs, in which one child “dictates” to the other a letter about a topic of his/her choice (e.g., birthday wishes to a classmate, farewell to a family member, and discussion of the school day with dad). This activity is functional in fostering children's conceptual knowledge of the specific forms that writing takes when we change the support on which we write. Children improve their awareness of the relationship between conventional rules about writing and the specific context (e.g., writing a letter or a birthday card).

Below, we provide an overview of the activities involved in PASSI; see Supplemental Materials for a more detailed description of the intervention with examples of activities.

##### Targeting the graphic sign

To improve the children's ability to graphically represent signs, we implemented activities such as: Creating shapes with cardboard or a rope; drawing shapes with chalk on the floor and then having the children walk on them; observing the shape of objects present in the children's everyday life and describing their perimeter; guessing hidden shapes by seeing only some detail; noticing that if we partially modify a sign, then the whole configuration of the sign changes as well; reflecting on the difference among real objects, objects in a picture, and drawn objects; reflecting on the different representations of the same object; identifying the essential traits to characterize an object through drawing; and understanding that the same graphical sign can be assigned different meanings if represented in a different position in relation to the context.

##### Targeting the orthographic sign

We implemented the following activities: Activities to familiarize the children with usual and unusual writing instruments; guessing games to discriminate written words from scribbles; activities in which the children played with letters; activities in which the children had to find letters within complex patterns; and activities in which the children had to read street signs.

##### Targeting the numeric sign

To help the children differentiate among different symbolic systems, we also targeted the writing of numbers. We implemented the following activities: Nursery rhymes in which the children associated the names of the numbers with their representation; games to associate the number with the symbolic sign; activities in which the children used written numbers to discriminate positions and quantities; and activities in which the children had to recognize numbers within complex patterns. We also constructed a clock to identify daily activities through the hours of the day and a thermometer with the line of numbers.

##### Control group

We asked the control group teachers to schedule the early literacy activities typically performed in the regular school curriculum in the same time slots while the experimental group children were working on conceptual knowledge of the writing system intervention for the same length of time and same frequency. More specifically, the control group worked on the following skills:

##### Grapho-motor skills

Playing with materials, transforming and creating with hands small and large objects, gluing and taping, cutting, filing, tracing contours, drawing straight, and curved lines, drawing labyrinths and paths, painting, coloring, and drawing repeated ornaments.

##### Literacy skills

Listening to and telling stories, inventing stories, illustrating stories, inventing nursery rhymes, playing with words (e.g., “which words do you know that begin with the letter …?”), recognizing initial and final phonemes in a word, reflecting on the length of words, segmenting and combining words in syllables and phonemes.

##### Sensorial skills

Discriminating the basic colors; mixing them to create new colors; discriminating sounds, rhythm, high, and low sounds; discriminating smooth and rough materials and soft and hard materials; discriminating flavors (sweet, sour, and savory), mixing water and flour to make bread or pizza; whipping cream and baking simple sweets; discriminating the smells of flowers, beverages, food, perfumes, and glue; and describing the differences.

### Measures

Invented Spelling (Bigozzi et al., 2016a,b)

The children were asked to write as best as they could the following words: their name, mum [*mamma*], dad [*babbo*], child [*bambino*], and little bird [*uccellino*]. The children's invented spelling of words was categorized into four sequential schemes: graphic scheme, pseudo-writing, symbolic scheme, and conventional spelling (see Table 2). Each item was coded, and a mean score was calculated. Participants' scores could range from a minimum of 1 to a maximum of 4. The reliability score was good, with α = 0.91. Two raters scored all the children's attempts. The inter-rater reliability score was strong, with k = 0.90. In the few instances when there was a discrepancy in their scoring, both scorers discussed each item until a consensus was reached.

**Table 2:** Coding system for the invented spelling of words task.

**Sequential scheme**	**Description**	**Example**
1. Graphic scheme	Scribbles	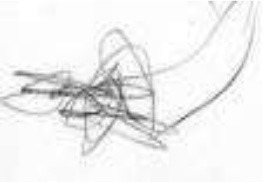
2. Pseudo-writing (first forms of writing)	Segmentation into units (spelling includes distinct units); Complexity (production of complex shapes, such as circles or triangles); variety (different shapes for the units produced)	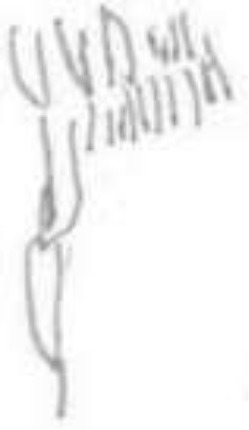
3. Symbolic scheme	Conventional symbol (the invented spelling includes at least one real letter); phonetic representation (the invented spelling includes at least one letter phonetically correlated with the word);	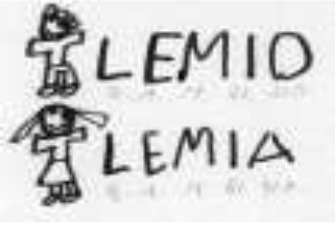
4. Conventional spelling	Conventional spelling of the word	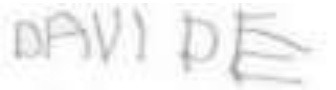

#### Invented spelling of numbers

The children were also asked to write all the numbers that they knew. Two independent raters attributed a global score to the children's production and categorized it into three sequential schemes following the previous coding scheme: graphic scheme, pseudo-writing, symbolic scheme, and conventional spelling (see Table 3). To attribute scores to the children's production, we adapted Yamagata's coding system 2007. Originally, Yamagata's coding system had three main categories and eight subcategories. Our first sequential scheme, the graphic scheme, corresponds to Yamagata's first category, graphic products (sub-categories 1 and 2). Our second sequential scheme, pseudo-writing, corresponds to Yamagata's second category, writing-like products (sub-categories 3, 4, 5, 6, and 7). We added a third sequential scheme, the symbolic scheme, to code children's productions that were similar to conventional spelling but differed from it in some detailed manner (e.g., the number “3” written with three humps). Finally, our fourth sequential scheme, conventional spelling, corresponds to Yamagata's third category, conventional products (sub-category 8). Two raters scored all the children's attempts. The inter-rater reliability score was strong, with k = 0.91. In the few instances when there was a discrepancy in their scoring, both scorers discussed each item until a consensus was reached.

**Table 3:** Coding system for the invented spelling of numbers task.

**Sequential scheme**	**Description**	**Example**
1. Graphic scheme	Scribbles and marks such as scrolled circles.	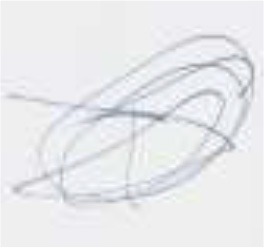
2. Pseudo-writing (first forms of writing)	Pseudo-writing of numbers, sharing some features of number writing, such as linearity, segmentation into units, and simple units repeated	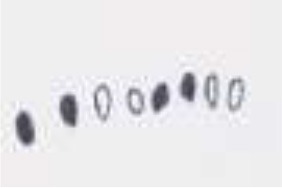
3. Symbolic scheme	Plausible numbers, ciphers similar to the conventional sign but that slightly diverge from it	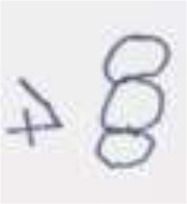
4. Conventional spelling	Conventional spelling of the number	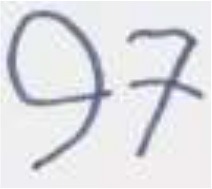

#### Knowledge of the alphabet (aram and biron, 2004)

The children were asked to recognize the letters of the alphabet from a set of 21 printed letters. One point was assigned for every letter correctly recognized, for a maximum of 21 points. The reliability score was good, with an α coefficient = 0.88.

#### Orthographic awareness (levy et al., 2006)

Twelve pairs of patterns of signs corresponding to words and non-words were represented on cardboard. In each pair, the non-word included a characteristic that violated the rules of the writing system (i.e., scribble, fonts like letters, figures, non-linearity, excessive spacing, one letter only, a mix of letters and numbers, the same letter repeated, letters written upside down, letters written backwards, only consonants, and only vowels). The children had to identify which stimulus corresponded to a word that could be read. For each word correctly identified, the children received one point, for a maximum of 12 points. The reliability score was good, with an α coefficient = 0.87.

#### Drawing skills

To understand if the child was able to communicate different types of information depending on the request, we assigned a drawing task with a “contrastive” instruction. The child had to draw a person standing still and then a person running. The children's productions were coded on the basis of the differences between the two drawings in several dimensions: head orientation; body orientation; feet orientation; the representation of elbows, arms, ankles, knees, hair, and clothes; and the distance between feet (Morra, 2005). The differentiation score can range between 0 and 11 points. Two raters scored all the children's attempts. The inter-rater reliability score was strong, with k = 0.95. In the few instances when there was a discrepancy in their scoring, both scorers discussed each item until a consensus was reached.

#### Visual-motor integration, VMI (beery and buktenica, 2000)

This test evaluates how children integrate their visual and motor skills by asking them to copy 18 geometrical shapes of increasing complexity. One point was assigned for every shape correctly copied. Scores could range between 0 and 18 points. The reliability score was good, with an α coefficient = 0.91. Two raters scored all of the children's attempts. The inter-rater reliability score was strong, with k = 0.87. In the few instances when there was a discrepancy in their scoring, both scorers discussed each item until a consensus was reached.

### Research design and data analysis

To test the hypotheses of this study, a parallel cluster randomized trial with a control group research design^1^ with pre-test and post-test comparisons between two groups was carried out (Campbell et al., 2004). The research design of this study followed all indications of the Declaration of Helsinki (World Medical Association, 2013) and was approved by the Ethics Committee of the Department of Psychology at the University of Florence, Italy. We collected the written informed consent forms from the participants' parents. We strictly adhered to the requirement of privacy required by Italian law. Six teachers (and their three respective classes) were randomly assigned to the experimental group and six to the control group. The two groups were assessed with the same tests in both the pre- and post-test stages and differed in that the experimental group received a 3-month invented spelling intervention, whereas the control group followed the regular curriculum. Given the nested nature of the data, the appropriate statistical procedures were applied.

The principal descriptive statistics (mean, standard deviation, skewness, and kurtosis coefficients) were calculated. We applied increasing monotonic transformations to all variables that were not normally distributed (Fox, 2008). Differences between post-test and pre-test performances were calculated for each variable and used as dependent variables. Because the study was a parallel “cluster” randomized trial with a control group, in which classes and their teachers were randomly assigned to the control or experimental condition, we analyzed the data using complex samples general linear model (GLM) analyses. Group was included as fixed factor, pre-test scores were included as covariates, and classroom as cluster variable.

## Results

### Descriptive statistics

The descriptive analyses for the experimental and control groups are presented in Table 4. On average, the children's conceptual knowledge of the writing system was between the pseudo-writing and symbolic schemes. The children were able to recognize an average of six letters, although the knowledge of letter performances was characterized by great variance. The children were able to recognize and discriminate from pseudo-words approximately half of the words presented. In contrast, drawing skills were quite low, with children being hardly able to discriminate between a running vs. a still person when drawing. Finally, the VMI performances were in line with what was expected of children of this age, with an average of half of the 18 geometrical shapes being correctly reproduced.

**Table 4:** Descriptive statistics for the total sample (*n* = 124), and for the experimental group (*N* = 69) vs. control group (*N* = 55).

**Variable**	**Total**				**Experimental**				**Control**			
	**Min**	**Max**	**M**	***SD***	**Min**	**Max**	**M**	***SD***	**Min**	**Max**	**M**	***SD***
Invented spelling words_1	1	4	2.73	0.69	1	4	2.82	0.65	1	3.75	2.62	0.72
Invented spelling numbers_1	1	3	2.60	0.52	1	3	2.55	0.53	1	3	2.67	0.51
Knowledge of letters_1	0	21	8.76	8.11	0	21	8.22	8.29	0	21	9.44	7.91
Orthographic awareness_1	0	12	7.42	3.48	0	12	7.03	3.40	0	12	7.91	3.56
Drawing_1	0	3	0.71	0.88	0	3.16	0.70	0.88	0	3	0.72	0.88
Visual-Motor Integration_1	0	18	11.04	4.71	0	18	11.19	5.18	1	18	10.85	4.07
Invented spelling words_2	1	4	2.88	0.67	1	4	3.02	0.60	1	3.75	2.70	0.72
Invented spelling numbers_2	2	3	2.65	0.48	2	3	2.67	0.48	2	3	2.62	0.49
Knowledge of letters_2	0	21	10.66	8.05	0	21	11.23	8.07	0	21	9.95	8.04
Orthographic awareness_2	0	12	8.81	2.56	4	12	9.39	1.86	0	12	8.09	3.11
Drawing_2	0	9	1.44	1.85	0	9	1.46	1.95	0	7	1.40	1.73
Visual-Motor Integration_2	1	18	11.73	3.99	4	18	12.32	4.07	1	18	11	3.79
Invented spelling words_difference	−1	1	0.04	0.35	0	1	0.12	0.32	−1	1.50	0.05	0.39
Invented spelling numbers_difference	−1	1.5	0.12	0.37	−0.5	1	0.17	0.34	−1	1	−0.06	0.36
Knowledge of letters_difference	−3	12	1.90	2.86	−1	12	3.02	3.10	−3	8	0.51	1.70
Orthographic awareness_difference	−6	10	1.40	2.72	−4	10	2.36	2.75	−6	7	0.18	2.14
Drawing_difference	−7	6	0.16	1.99	−6	6	0.20	2.21	−7	4	0.11	1.70
Visual-Motor Integration_difference	−5	11	0.69	2.37	−3	11	1.13	2.39	−5	11	0.15	2.26

All variables included in the study correlated with each other, except for drawing in the pre-test and the invented spelling of words in the post-test, drawing in the pre-test and knowledge of letters in the post-test, in addition to drawing between pre-test and post-test (see Table 5). These results confirm the stability of the emergent literacy construct, which includes several interconnected literacy-related skills (Lonigan et al., 2000).

**Table 5:** Correlation between dependent variables.

	**Variable**	**1**	**2**	**3**	**4**	**5**	**6**	**7**	**8**	**9**	**10**	**11**	**12**
1	Invented spelling words_1	1											
2	Invented spelling numbers_1	0.62^**^	1										
3	Knowledge of letters_1	0.52^**^	0.56^**^	1									
4	Orthographic awareness_1	0.52^**^	0.61^**^	0.58^**^	1								
5	Drawing_1	0.29^**^	0.24^**^	0.30^**^	0.35^**^	1							
6	Visual-Motor Integration_1	0.66^**^	0.67^**^	0.62^**^	0.53^**^	0.35^**^	1						
7	Invented spelling words_2	0.80^**^	0.63^**^	0.49^**^	0.45^**^	0.23^**^	0.61^**^	1					
8	Invented spelling numbers_2	0.63^**^	0.76^**^	0.62^**^	0.61^**^	0.28^**^	0.73^**^	0.61^**^	1				
9	Knowledge of letters_2	0.53^**^	0.54^**^	0.94^**^	0.57^**^	0.24^**^	0.62^**^	0.52^**^	0.65^**^	1			
10	Orthographic awareness_2	0.62^**^	0.42^**^	0.42^**^	0.63^**^	0.29^**^	0.56^**^	0.53^**^	0.55^**^	0.47^**^	1		
11	Drawing_2	0.38^**^	0.31^**^	0.37^**^	0.20^**^	0.40^**^	0.42^**^	0.30^**^	0.33^**^	0.35^**^	0.26^**^	1	
12	Visual-Motor Integration_2	0.67^**^	0.70^**^	0.62^**^	0.55^**^	0.32^**^	0.86^**^	0.69^**^	0.70^**^	0.64^**^	0.54^**^	0.42^**^	1

*1, pre-test; 2, post-test*.

** *p < 0.01*.

### Pre-test differences

Before comparing the effect of the intervention on the students' gains over time, we controlled for baseline equivalence on the pre-test measures. The two groups did not differ in any measure: the invented spelling of words [*t* = −1.630, *df* = 122, *p* = 0.11; 95%CI = −0.044; 0.444], the invented spelling of numbers [*t* = −1.294, *df* = 122, *p* = 0.198; 95%CI = −0.309; 0.065], knowledge of letters [*t* = −0.830, *df* = 122, *p* = 0.408; 95%CI = −4.126; 1.688], orthographic awareness [*t* = −1.403, *df* = 122, *p* = 0.163; 95%CI = −0.339; 0.294], drawing [*t* = −0.140, *df* = 122, *p* = 0.889; 95%CI = −0.339; 0.294], or VMI [*t* = 0.391, *df* = 122, *p* = 0.696; 95%CI = −1.356; 2.024].

### Effects of intervention on dependent variables

According to the results from the complex samples GLMs, group significantly explained differences in growth from pre-test to post-test performances in the following variables: invented spelling of words, knowledge of letters, and orthographic awareness. Growth in orthographic awareness was also explained by pre-test performances in knowledge of letters and visual-motor integration. The effect sizes of the complex samples GLMs were moderate for orthographic awareness and knowledge of letters, and low for invented spelling of words, invented spelling of numbers, drawing, and visual-motor integration (see Table 6).

**Table 6:** Results from the complex samples GLM analyses with differences between post-test and pre-test scores as dependent variables, group as factor, pre-test scores as covariates, and classroom as cluster variable.

**Dependent Variables**	**Parameters**	**df**	**Wald's *F***	***p***	**95%CI**	***t***	***p***
Invented spelling of words [R^2^ = 0.05]	Group	1, 5	10.15	0.02	0.02; 0.21	3.19	0.02
	Knowledge of letters	1, 5	0.14	0.73	−0.01; 0.01	0.37	0.73
	Orthographic awareness	1, 5	1.12	0.34	−0.05; 0.02	−1.06	0.34
	Visual–Motor Integration	1, 5	0.05	0.83	−0.02; 0.03	0.22	0.83
	Drawing	1, 5	2.12	0.21	−0.09; 0.03	−1.46	0.21
	Invented spelling of numbers	1, 5	0.27	0.62	−0.26; 0.40	0.52	0.62
Invented spelling of numbers [R^2^ = 0.09]	Group	1, 5	5.52	0.07	−0.02; 0.39	2.35	0.07
	Invented spelling of words	1, 5	2.81	0.15	−0.22; 0.05	−1.68	0.15
	Knowledge of letters	1, 5	0.58	0.48	−0.01; 0.02	0.76	0.48
	Orthographic awareness	1, 5	0.52	0.51	−0.04; 0.02	−0.72	0.51
	Drawing	1, 5	0.10	0.77	−0.12; 0.15	0.31	0.77
	Visual-Motor Integration	1, 5	0.12	0.74	−0.03; 0.04	0.35	0.74
Knowledge of letters [R^2^ = 0.23]	Group	1, 5	165.95	< 0.01	2.10; 3.14	12.88	< 0.01
	Invented spelling of words	1, 5	0.55	0.49	−2.04; 1.13	−0.74	0.49
	Invented spelling of numbers	1, 5	0.02	0.89	−2.66; 2.37	−0.15	0.89
	Orthographic awareness	1, 5	0.78	0.42	−0.16; 0.33	0.89	0.42
	Drawing	1, 5	4.58	0.09	−1.51; 0.14	−2.14	0.09
	Visual-Motor Integration	1, 5	1.22	0.32	−0.08; 0.20	1.11	0.32
Orthographic awareness [R^2^ = 0.35]	Group	1, 5	10.50	0.02	0.34; 2.97	3.24	0.02
	Invented spelling of words	1, 5	1.00	0.36	−1.05; 2.39	1.00	0.36
	Invented spelling of numbers	1, 5	3.98	0.10	−4.77; 0.60	−2.00	0.10
	Knowledge of letters	1, 5	8.61	0.03	−0.18; −0.01	−2.93	0.03
	Drawing	1, 5	4.70	0.08	−0.72; 0.06	−2.17	0.08
	Visual-Motor Integration	1, 5	10.84	0.02	0.03; 0.21	3.29	0.02
Drawing [R^2^ = 0.07]	Group	1, 5	0.12	0.74	−0.71; 0.54	−0.35	0.74
	Invented spelling of words	1, 5	1.70	0.25	−0.35; 1.08	1.31	0.25
	Invented spelling of numbers	1, 5	1.30	0.31	−0.49; 1.26	1.14	0.31
	Knowledge of letters	1, 5	2.66	0.16	−0.01; 0.06	1.63	0.16
	Orthographic awareness	1, 5	5.40	0.07	−0.42; 0.02	−2.32	0.07
	Visual-Motor Integration	1, 5	0.05	0.84	−0.09; 0.11	0.22	0.84
Visual-Motor Integration [R^2^ = 0.10]	Group	1, 5	4.07	0.10	−0.29; 2.41	2.02	0.10
	Invented spelling of words	1, 5	0.77	0.42	−2.24; 1.10	−0.88	0.42
	Invented spelling of numbers	1, 5	0.02	0.90	−2.01; 2.23	0.14	0.90
	Knowledge of letters	1, 5	1.34	0.30	−0.08; 0.03	−1.16	0.30
	Orthographic awareness	1, 5	0.26	0.63	−0.11; 0.16	0.51	0.63
	Drawing	1, 5	2.23	0.20	−0.83; 0.22	−1.49	0.20

## Discussion

This study tested the efficacy of PASSI, an intervention targeting 3- to 5-year-old children's conceptual knowledge of the Italian writing system, in enhancing early literacy skills. The results partially confirmed the research hypothesis, in line with prior studies on this construct (Silva and Martins, 2003; Ouellette and Sénéchal, 2008; Rieben et al., 2009; Levin and Aram, 2013). Overall, the interaction between group and time was significant for all emergent literacy skills, confirming that this set of early skills can be enhanced through interventions (Bus and van Ijzendoorn, 1999; Justice and Pullen, 2003).

More specifically, PASSI was effective in improving both, conceptual knowledge of the writing system (as assessed by the invented spelling of words task), and literacy-related skills (i.e., knowledge of the alphabet and orthographic awareness). This result has practical implications due to the relevance of children's conceptual knowledge of the writing system for the acquisition of reading and spelling (Ouellette and Sénéchal, 2017) and for the prediction of related disorders (Bigozzi et al., 2016a). PASSI aims at triggering children's metalinguistic reflection on the writing system by giving them an insight into its structure (Treiman, 1998), also granting to teachers insight into children's conceptual knowledge of the writing system. More specifically, PASSI intervenes in the simultaneous integration of the dual code, decoding, and coding, in three different symbolic systems (word writing, number writing, and drawing). PASSI does so through an embedded-explicit approach in which teachers target specific subskills (reflection on the graphic, symbolic and phonological aspect of written signs) and emphasize children's contextualized interactions with oral and written language (Justice and Kaderavek, 2004). PASSI targets children's phonological awareness by improving the integration between children's skills in this construct and other related emergent literacy skills, in light of the limited role that phonological awareness plays in transparent writing systems (Ziegler and Goswami, 2005; Notarnicola et al., 2012; Bigozzi et al., 2016a; Daniels and Share, 2018; Diamanti et al., 2018). As suggested by several theories (e.g., psycholinguistic grain size theory), the consistency of spelling–sound mappings modulate the importance of the role of phonological skills in reading and spelling acquisition in transparent orthographies (Ziegler and Goswami, 2005; Bigozzi et al., 2017; Daniels and Share, 2018; Diamanti et al., 2018).

However, similar to prior studies on invented spelling interventions, the impact of PASSI on improving children's conceptual knowledge of the writing system was small (Silva and Martins, 2003; Ouellette and Sénéchal, 2008; Rieben et al., 2009; Levin and Aram, 2013). Of notice, although on a descriptive level the experimental group showed an increment in performance in the invented spelling of numbers task, whereas the control group showed a decrease in the same task, the analysis did not reach the conventional threshold for significance. These data confirm that emergent literacy and numeracy are not overlapping domains but present some differences (Tolchinsky Landsmann, 2003) and can be explained by a few hypotheses. First, the result could depend on the fact that the conventional use of numbers appears developmentally earlier and more frequently than the conventional use of letters (Yamagata, 2007). Alternatively, the invented spelling tasks might have been too easy for the children, as shown by the means in both conditions, which might have led to underestimation of the benefit of the treatment. Or, the result might depend on the design of the invented spelling of numbers task. We chose to assign a global score to children's performances, but children may have known more numbers in time 2 than they did in time 1. Future studies should replicate the design of this study with an improved version of the invented spelling of numbers task. Finally, whereas prior studies on emergent literacy interventions have confirmed that their efficacy is domain-specific (e.g., Lonigan et al., 2013), previous studies showed that broader teaching approaches provided by parents on literacy (e.g. direct teaching of literacy skills) promoted counting skills too (LeFevre et al., 2009; e.g., Anders et al., 2012; Manolitsis et al., 2013). Thus, results might depend on the domain-specific nature of PASSI.

Interestingly, the efficacy of PASSI was higher for growth in knowledge of letters and orthographic awareness, relevant skills developing before the onset of formal skills and connected to reading and spelling acquisition (Silva and Martins, 2003; Treiman et al., 2007a,b, 2015, 2016; Ouellette and Sénéchal, 2008; Rieben et al., 2009; Puranik et al., 2011; Levin and Aram, 2013). Knowledge of alphabet was measured through a letter recognition task, rather than with a letter writing task. Prior studies have emphasized the importance of letter writing for future spelling acquisition (Puranik et al., 2013), however in this study we were interested in children's emerging knowledge about letters. It is important to notice that this task posits different cognitive demands as compared to the other tasks involving writing, a difference that may influence results. Rather than a related skill, orthographic awareness represents an important component of children's conceptual knowledge of the writing system, which is systematically interacting with phonological awareness in their attempts at spelling (Adams, 1998; Ouellette and Sénéchal, 2008).

Conversely, PASSI did not contribute to improving the children's drawing or visual-motor integration skills, which confirms the specificity of this intervention and emphasizes how, developmentally, drawing and writing skills are already separate domains in the child (Tolchinsky Landsmann and Karmiloff-Smith, 1992). Of notice, children's visual-motor integration skills were involved with children's orthographic awareness, confirming the involvement of domain-general skills in the application and execution of knowledge on phonological-orthographic connectivity (Pinto and Camilloni, 2012; Read and Treiman, 2013).

For these reasonsresults from this study are in accordance with Levin and Aram (2013) criticism of prior invented spelling interventions that designed developmentally tailored interventions (Silva and Martins, 2003; Ouellette and Sénéchal, 2008), which could constrain children's progress. Concerning the role played by children's visual-motor skills, while our data confirm the involvement of this bottom-up construct in children's emergent literacy skills (Pinto and Camilloni, 2012), the fact that the group explained most of the variance of the dependent variables, even after the effect of children's visual-motor integration skills were checked, confirms that children rely on other sources of information when “inventing spelling,” namely, their knowledge of the structure of the writing system.

### Limitations and directions for future research

Although children's emergent attempts at spelling must be considered in light of the characteristics of their writing system (Levin and Aram, 2013; Read and Treiman, 2013) and its transparency (Ziegler et al., 2010), the efficacy of PASSI might be extended to other languages. Previous studies have confirmed the efficacy of invented spelling interventions in both transparent (e.g., Portuguese, Silva and Martins, 2003) and opaque orthographies (e.g., French, Ouellette and Sénéchal, 2008; Rieben et al., 2009; or Hebrew, Levin and Aram, 2013). We speculate that the embedded component of PASSI, in which children's spontaneous interactions with the symbolic systems included in their environment are emphasized and supported, might be cross-linguistically similar, whereas the explicit component of PASSI, which includes children's engagement with grapheme-phoneme correspondences, might be language bound and should be adapted when used in different contexts. Future studies should confirm these speculations on the generalizability of PASSI to other languages. Similar to other studies on invented spelling interventions (Silva and Martins, 2003; Ouellette and Sénéchal, 2008; Rieben et al., 2009; Levin and Aram, 2013), the efficacy of PASSI on conceptual knowledge of the writing system was only moderate. This similarity in results suggests an apparent difficulty in enhancing children's conceptual knowledge of the writing system through intervention. One reason might be the multicomponential nature of this construct, which means that an intervention needs to target each of these components (e.g., phonological awareness, orthographic awareness, and visual-motor integration) and integration among them (e.g., sound-sign mapping). Consequently, for students, it might be difficult to transfer what was learned during the intervention to other tasks. An indirect confirmation derives from the fact that Ouellette and Sénéchal's intervention 2008 did not improve the reading performance of words not included in the intervention, a datum that suggests that the intervention was not effective in targeting children's grapho-phonemic mapping skills (Levin and Aram, 2013). A second reason might be the difficulty that children face in differentiating between different symbolic systems, for example, drawing and writing. Frequently, schools adopt a mixed writing and drawing approach in kindergarten, in which children are asked to write a word in response to the associated picture. Such a practice should be reconsidered, given that prior studies have shown that mixing these two systems might retard automaticity in writing (Adi-Japha and Freeman, 2001), a phenomenon that might have contributed to reducing the efficacy of PASSI.

Results from the present study are limited to tasks adopted to measure emergent-literacy skills, and the effect of control variables included, namely drawing and visual-motor integration skills. The effect of PASSI on growth in invented spelling was small for words and non-significant for numbers, but results might depend on the characteristics of the tasks (e.g., limited number of items for invented spelling of words, or absence of a specific end for invented spelling of numbers). In terms of control variables, future studies should verify whether other developmentally relevant skills moderate the beneficial effects of PASSI (e.g., mental state talk, given its context-dependency, Pinto et al., 2016).

In this study, teachers, not students, were randomly assigned to conditions. We controlled the effect of data nested within these clusters statistically through a complex samples GLM approach. However, given the lack of a true experimental control in the clustered design employed in this study, we could only produce evidence supporting the efficacy of the PASSI intervention, which should be “confirmed” or “verified” through future studies employing a randomized trial research design.

### Implications for applied practice

Prior studies have emphasized the importance of children's conceptual knowledge of the writing system as a specific predictor of reading and spelling disorders (Bigozzi et al., 2016a). This predictor was assessed in kindergarten, before the onset of formal literacy. This study supports the hypothesis that this important process can be fostered through a targeted intervention. This result has relevant practical implications, given that it suggests that teachers can act on a specific risk factor of reading and spelling disorders when children are still in kindergarten, contributing to preventing them or at least reducing their severity through a primary prevention approach. PASSI is not an intervention promoting early learning to read and spell in a formal manner; rather, it fosters emergent literacy processes associated with literacy acquisition. Thus, PASSI is an “ecological” instrument that can be integrated in kindergarten's daily routines. Moreover, it is crucial to implement early interventions supporting children spelling acquisition, so that decoding and coding processes are automatized by children before they become functional to more complex tasks, such as composing (Pinto et al., 2015b) or reflecting (Bigozzi et al., 2011).

## Conclusion

In conclusion, this study confirms the validity of PASSI, an intervention into children's conceptual knowledge of the writing system targeting children's emergent literacy skills through an embedded-explicit approach (Justice and Kaderavek, 2004), rather than through tailored interventions (Levin and Aram, 2013). This emergent literacy construct is a cognitive precursor of reading and spelling acquisition (Bigozzi et al., 2016a,b; Pinto et al., 2017). PASSI promoted engagement with the graphic, orthographic and numeric sign by emphasizing children's daily self-initiated, naturalistic, and contextualized interactions with oral and written language, in addition to implementing structured, sequenced and directed instruction targeting specific skills. In this manner, the children were able to develop specific emergent literacy skills, such as knowledge of letters and orthographic awareness, and an integrated conceptual knowledge of the writing system, all of which are significantly associated to later reading and spelling development (Puranik et al., 2011).

## Author contributions

All authors listed have made a substantial, direct and intellectual contribution to the work, and approved it for publication.

### Conflict of interest statement

The authors declare that the research was conducted in the absence of any commercial or financial relationships that could be construed as a potential conflict of interest.

## Footnotes

^1^Groups of individuals (school classes in this study), rather than individuals, are randomly assigned to study groups (Campbell et al., 2004).

## References

[B1] Adams M. (1998). Beginning to Read: Thinking and Learning about Print. Cambridge: MIT Press.

[B2] Adi-Japha E. Freeman N. H. (2001). Development of differentiation between writing and drawing systems. Dev. Psychol. 37, 101–114. 10.1037/0012-1649.37.1.101 11206425

[B3] Anders Y. Rossbach H.-G. Weinert S. Ebert S. Kuger S. Lehrl S. (2012). Home and preschool learning environments and their relations to the development of early numeracy skills. Early Child. Res. Q. 27, 231–244. 10.1016/J.ECRESQ.2011.08.003

[B4] Aram D. Biron S. (2004). Joint storybook reading and joint writing interventions among low SES preschoolers: differential contributions to early literacy. Early Child. Res. Q. 19, 588–610. 10.1016/j.ecresq.2004.10.003

[B5] Aram D. Levin I. (2004). The role of maternal mediation of writing to kindergartners in promoting literacy in school: a longitudinal perspective. Read. Writ. 17, 387–409. 10.1023/B:READ.0000032665.14437.e0

[B6] Beery K. E. Buktenica N. E. (2000). Developmental Test of Visual-Motor Integration. Firenze, IT: Giunti O.S.

[B7] Berninger V. W. Nielsen K. H. Abbott R. D. Wijsman E. Raskind W. (2008). Writing problems in developmental dyslexia: under-recognized and under-treated. J. Sch. Psychol. 46, 1–21. 10.1016/j.jsp.2006.11.008 18438452 PMC2344144

[B8] Bigozzi L. Tarchi C. Caudek C. Pinto G. (2016a). Predicting reading and spelling disorders: a 4-year cohort study. Front. Psychol. 7:337. 10.3389/fpsyg.2016.00337 27014145 PMC4783383

[B9] Bigozzi L. Tarchi C. Pezzica S. Pinto G. (2016b). Evaluating the predictive impact of an emergent literacy model on dyslexia in Italian children: a four-year prospective cohort study. J. Learn. Disabil. 49, 51–65. 10.1177/0022219414522708 24608754

[B10] Bigozzi L. Tarchi C. Pinto G. (2017). Consistency and stability of italian children's spelling in dictation versus composition assessments. Read. Writ. Q. 33, 109–122. 10.1080/10573569.2015.1102111

[B11] Bigozzi L. Vezzani C. Tarchi C. Fiorentini C. (2011). The role of individual writing in fostering scientific conceptualization. Eur. J. Psychol. Educ. 26, 45–59. 10.1007/s10212-010-0031-8

[B12] Bus A. G. van Ijzendoorn M. H. (1999). Phonological awareness and early reading: a meta-analysis of experimental training studies. J. Educ. Psychol. 91, 403–414. 10.1037/0022-0663.91.3.403

[B13] Campbell M. K. Elbourne D. R. Altman D. G. (2004). Consort statement: extension to cluster randomised trials. BMJ 328, 702–708. 10.1136/bmj.328.7441.702. 15031246 PMC381234

[B14] Caravolas M. Lervåg A. Mousikou P. Efrim C. Litavsky M. Onochie-Quintanilla E.. (2012). Common patterns of prediction of literacy development in different alphabetic orthographies. Psychol. Sci. 23, 678–686. 10.1177/0956797611434536 22555967 PMC3724272

[B15] Cossu G. Gugliotta M. Marshall J. C. (1995). Acquisition of reading and written spelling in a transparent orthography: two non parallel processes? Read. Writ. 7, 9–22. 10.1007/BF01026945

[B16] Daniels P. T. Share D. L. (2018). Writing system variation and its consequences for reading and dyslexia. Sci. Stud. Read. 22, 101–116. 10.1080/10888438.2017.1379082

[B17] Diamanti V. Goulandris N. Campbell R. Protopapas A. (2018). Dyslexia profiles across orthographies differing in transparency: an evaluation of theoretical predictions contrasting english and greek. Sci. Stud. Read. 22, 55–69. 10.1080/10888438.2017.1338291.

[B18] Ferreiro E. (1988). L'e'criture avant la lettre (En. tr. Writing before reading), in La Prodution des Notations chez le Jeune Enfant (En. tr. Notational Production in Young Children), ed Sinclair H. (Paris: Presses Universitaires de France), 18–69.

[B19] Fox J. (2008). Applied Regression Analysis and Generalized Models. Thousand Oaks, CA: SAGE Publications.

[B20] Freeman N. H. (1987). Current problems in the development of representational picture-production. Arch. Psychol. 55, 127–152.

[B21] Furnes B. Samuelsson S. (2010). Predicting reading and spelling difficulties in transparent and opaque orthographies: a comparison between Scandinavian and US/Australian children. Dyslexia 16, 119–142. 10.1002/dys.401 20440743 PMC2908032

[B22] Germano G. D. Reilhac C. Capellini S. A. Valdois S. (2014). The phonological and visual basis of developmental dyslexia in Brazilian Portuguese reading children. Front. Psychol. 5:1169. 10.3389/fpsyg.2014.01169 25352822 PMC4196516

[B23] Goodnow J. J. Levine R. A. (1973). The grammar of action”: sequence and syntax in children's copying. Cogn. Psychol. 4, 82–98. 10.1016/0010-0285(73)90005-4

[B24] Justice L. M. Kaderavek J. N. (2004). Embedded-explicit emergent literacy intervention I: background and description of approach. Lang. Speech Hear. Serv. Sch. 35, 201–211. 10.1044/0161-1461(2004/020) 15248791

[B25] Justice L. M. Pullen P. C. (2003). Promising interventions for promoting emergent literacy skills: three evidence-based approaches. Topics Early Child. Spec. Educ. 23, 99–113. 10.1177/02711214030230030101

[B26] Katz L. Frost R. (1992). The reading process is different for different orthographies: the orthographic depth hypothesis, in Orthography, Phonology, Morphology and Meaning, eds Frost R. Katz L. (Amsterdam, NL: North-Holland), 67–84.

[B27] Landerl K. Ramus F. Moll K. Lyytinen H. Leppänen P. H. T. Lohvansuu K.. (2013). Predictors of developmental dyslexia in European orthographies with varying complexity. J. Child Psychol. Psychiatry 54, 686–694. 10.1111/jcpp.12029 23227813

[B28] LeFevre J.-A. Skwarchuk S.-L. Smith-Chant B. L. Fast L. Kamawar D. Bisanz J. (2009). Home numeracy experiences and children's math performance in the early school years. Can. J. Behav. Sci. Can. des Sci. Comport. 41, 55–66. 10.1037/a0014532

[B29] Levin I. Aram D. (2013). Promoting early literacy via practicing invented spelling: a comparison of different mediation routines. Read. Res. Q. 48, 221–236. 10.1002/rrq.48

[B30] Levin I. Bus A. G. (2003). How is emergent writing based on drawing? Analyses of children's products and their sorting by children and mothers. Dev. Psychol. 39, 891–905. 10.1037/0012-1649.39.5.891 12952401

[B31] Levy B. A. Gong Z. Hessels S. Evans M. A. Jared D. (2006). Understanding print: early reading development and the contributions of home literacy experiences. J. Exp. Child Psychol. 93, 63–93. 10.1016/j.jecp.2005.07.003 16140318

[B32] Liberman I. Y. (1971). Basic research in speech and lateralization of language: some implications for reading disability. Bull. Ort. Soc. 21, 72–87. 10.1007/BF02663712

[B33] Lonigan C. J. Burgess S. R. Anthony J. L. (2000). Development of emergent literacy and early reading skills in preschool children: evidence from a latent-variable longitudinal study. Dev. Psychol. 36, 596–613. 10.1037/0012-1649.36.5.596 10976600

[B34] Lonigan C. J. Purpura D. J. Wilson S. B. Walker P. M. Clancy-Menchetti J. (2013). Evaluating the components of an emergent literacy intervention for preschool children at risk for reading difficulties. J. Exp. Child Psychol. 114, 111–130. 10.1016/j.jecp.2012.08.010 23073367 PMC3724170

[B35] Manolitsis G. Georgiou G. K. Tziraki N. (2013). Examining the effects of home literacy and numeracy environment on early reading and math acquisition. Early Child. Res. Q. 28, 692–703. 10.1016/J.ECRESQ.2013.05.004

[B36] Martins M. A. Silva C. (2006). The impact of invented spelling on phonemic awareness. Learn. Instr. 16, 41–56. 10.1016/j.learninstruc.2005.12.005

[B37] Morra S. (2005). Cognitive aspects of change in drawings: a neo-Piagetian theoretical account. Br. J. Dev. Psychol. 23, 317–341. 10.1348/026151005X27182

[B38] Notarnicola A. Angelelli P. Judica A. Zoccolotti P. (2012). Development of spelling skills in a shallow orthography: the case of Italian language. Read. Writ. 25, 1171–1194. 10.1007/s11145-011-9312-0

[B39] O'Donnell C. L. (2008). Defining, conceptualizing, and measuring fidelity of implementation and its relationship to outcomes in K-12 curriculum intervention research. Rev. Educ. Res. 78, 33–84. 10.3102/0034654307313793

[B40] Ouellette G. Sénéchal M. (2008). Pathways to literacy: a study of invented spelling and its role in learning to read. Child Dev. 79, 899–913. 10.1111/j.1467-8624.2008.01166.x 18717897

[B41] Ouellette G. Sénéchal M. (2017). Invented spelling in kindergarten as a predictor of reading and spelling in Grade 1: a new pathway to literacy, or just the same road, less known? Dev. Psychol. 53, 77–88. 10.1037/dev0000179 27617354

[B42] Paulesu E. Démonet J. F. Fazio F. McCrory E. Chanoine V. Brunswick N.. (2001). Dyslexia: cultural diversity and biological unity. Science 291, 2165–2167. 10.1126/science.1057179 11251124

[B43] Pinto G. Bigozzi L. Tarchi C. Accorti Gamannossi B. Canneti L. (2015a). Cross-lag analysis of longitudinal associations between primary school students' writing and reading skills. Read. Writ. 28, 1–23. 10.1007/s11145-015-9569-9.

[B44] Pinto G. Bigozzi L. Vezzani C. Tarchi C. (2017). Emergent literacy and reading acquisition: a longitudinal study from kindergarten to primary school. Eur. J. Psychol. Educ. 32, 571–587. 10.1007/s10212-016-0314-9

[B45] Pinto G. Camilloni M. (2012). The emergence of writing ability: drawing and writing differentiating processes, in Issues in Writing Research. In Honour of Piero Boscolo, eds Gelati C. Arfé B. Mason L. (Padova, IT: CLEUP), 29–44.

[B46] Pinto G. Tarchi C. Accorti Gamannossi B. Bigozzi L. (2016). Mental state talk in children's face-to-face and telephone narratives. J. Appl. Dev. Psychol. 44, 21–27. 10.1016/j.appdev.2016.02.004

[B47] Pinto G. Tarchi C. Bigozzi L. (2015b). The relationship between oral and written narratives: a three-year longitudinal study of narrative cohesion, coherence, and structure. Br. J. Educ. Psychol. 85, 551–569. 10.1111/bjep.12091 26373247

[B48] Puranik C. S. Lonigan C. J. Kim Y. S. (2011). Contributions of emergent literacy skills to name writing, letter writing, and spelling in preschool children. Early Child. Res. Q. 26, 465–474. 10.1016/j.ecresq.2011.03.002 21927537 PMC3172137

[B49] Puranik C. S. Petscher Y. Lonigan C. J. (2013). Dimensionality and reliability of letter writing in 3- to 5-year-old preschool children. Learn. Individ. Differ. 28, 133–141. 10.1016/j.lindif.2012.06.011 26346443 PMC4557880

[B50] Ravid D. Tolchinsky Landsmann L. (2002). Developing linguistic literacy: a comprehensive model. J. Child Lang. 29, 419–448. 10.1017/S0305000902005111 12109379

[B51] Read C. (1971). Pre-school children's knowledge of English phonology. Harv. Educ. Rev. 41, 1–34.

[B52] Read C. Treiman R. (2013). Children's invented spelling: what we have learned in forty years, in Rich Languages from Poor Inputs, eds Piattelli-Palmarini M. Berwick R. C. (Oxford: Oxford University Press), 195–209.

[B53] Rieben L. Ntamakiliro L. Gonthier B. Fayol M. Fayol M. (2009). Effects of various early writing practices on reading and spelling effects of various early writing practices on reading and spelling. Sci. Stud. Read. 9, 145–166. 10.1207/s1532799xssr0902

[B54] Seymour P. H. K. Aro M. Erskine J. M. (2003). Foundation literacy acquisition in European orthographies. Br. J. Psychol. 94, 143–174. 10.1348/000712603321661859 12803812

[B55] Silva C. Martins M. A. (2003). Relations between children's invented spelling and the development of phonological awareness. Educ. Psychol. 23, 3–16. 10.1080/01443410303218

[B56] Spira E. G. Bracken S. S. Fischel J. E. (2005). Predicting improvement after first-grade reading difficulties: the effects of oral language, emergent literacy, and behavior skills. Dev. Psychol. 41, 225–234. 10.1037/0012-1649.41.1.225 15656751

[B57] Sprenger-Charolles L. Siegel L. S. Jiménez J. E. Ziegler J. C. (2011). Prevalence and reliability of phonological, surface, and mixed profiles in dyslexia: a review of studies conducted in languages varying in orthographic depth. Sci. Stud. Read. 15, 498–521. 10.1080/10888438.2010.524463

[B58] Tarchi C. Pinto G. (2015). Educational practices and peer-assisted learning: analyzing students' interactive dynamics in a joint drawing task. Soc. Psychol. Educ. 18, 393–409. 10.1007/s11218-014-9269-3

[B59] Tolchinsky Landsmann L. (2003). The Cradle of Culture and What Children Know about Writing and Numbers Before Being Taught. Mahwah, NJ: Erlbaum.

[B60] Tolchinsky Landsmann L. Karmiloff-Smith A. (1992). Children's understanding of notations as domains of knowledge versus referential-communicative tools. Cogn. Dev. 7, 287–300. 10.1016/0885-2014(92)90017-L

[B61] Treiman R. (1998). Why spelling? The benefits of incorporating spelling into beginning to reading instruction, in Word Recognition in Beginning Literacy, eds Metsala J. L. Ehri L. C. (London: Lawrence Erlbaum Associates), 289–313.

[B62] Treiman R. Cohen J. Mulqueeny K. Kessler B. Schechtman S. (2007a). Young children's knowledge about printed names. Child Dev. 78, 1458–1471. 10.1111/j.1467-8624.2007.01077.x 17883442

[B63] Treiman R. Hompluem L. Gordon J. Decker K. Markson L. (2016). Young children's knowledge of the symbolic nature of writing. Child Dev. 70, 773–779. 10.1097/OGX.0000000000000256.Prenatal PMC4809759 26743133

[B64] Treiman R. Levin I. Kessler B. (2007b). Learning of letter names follows similar principles across languages: evidence from hebrew. J. Exp. Child Psychol. 96, 87–106. 10.1016/j.jecp.2006.08.002 17046016

[B65] Treiman R. Mulqueeny K. Kessler B. (2015). Young children's knowledge about the spatial layout of writing. Writ. Syst. Res. 7, 235–244. 10.1080/17586801.2014.924386 26366206 PMC4565506

[B66] Vernon S. Ferreiro E. (1999). Writing development: a neglegted variable in the consideration of phonological awareness. Harv. Educ. Rev. 69, 395–415. 10.17763/haer.69.4.p411667586738x0w

[B67] Whitehurst G. J. Lonigan C. J. (1998). Child development and emergent literacy. Child Dev. 69, 848–872. 10.1111/j.1467-8624.1998.tb06247.x 9680688

[B68] Wimmer H. Mayringer H. (2002). Dysfluent reading in the absence of spelling difficulties: a specific disability in regular orthographies. J. Educ. Psychol. 94, 272–277. 10.1037/0022-0663.94.2.272

[B69] World Medical Association (2013). Declaration of Helsinki: Ethical Principles for Medical Research Involving Human Subjects. Fortaleza. Available online at: http://www.wma.net/en/30publications/10policies/b3/index.html 10.1001/jama.2013.281053 24141714

[B70] Yamagata K. (2007). Differential emergence of representational systems: drawings, letters, and numerals. Cogn. Dev. 22, 244–257. 10.1016/j.cogdev.2006.10.006

[B71] Ziegler J. C. Bertrand D. Tóth D. Csépe V. Reis A. Faísca L.. (2010). Orthographic depth and its impact on universal predictors of reading: a cross-language investigation. Psychol. Sci. 21, 551–559. 10.1177/0956797610363406 20424101

[B72] Ziegler J. C. Goswami U. (2005). Reading acquisition, developmental dyslexia, and skilled reading across languages: a psycholinguistic grain size theory. Psychol. Bull. 131, 3–29. 10.1037/0033-2909.131.1.3 15631549

