# Supplementary material for: Improving Conceptual Knowledge of the Italian Writing System in Kindergarten: A Cluster Randomized Trial
Source: Front Psychol. 2018 Aug 7;9:1396. doi: 10.3389/fpsyg.2018.01396 (PMC6090480; doi:10.3389/fpsyg.2018.01396)
Supplement: Supplementary file 1 [file Table_1.DOCX]

Supplementary Material

Improving conceptual knowledge of the writing system in kindergarten: A cluster randomized trial

Giuliana Pinto, Lucia Bigozzi, Christian Tarchi^*^, Monica Camilloni

*** Correspondence:** Christian Tarchi: christian.tarchi@unifi.it

# PASSI (*Promoting the Achievement of Sound-Sign Integration*)

This supplemental material describes all the activities given to children as part of the PASSI intervention.

| **Targeting the graphic sign** | | | |
| --- | --- | --- | --- |
| **Activities** | **Objective** | | **Example of task** |
| **Decoding** |  | |  |
| A.1 Touch the sign  [3 tasks] | Identifying similarities and symbolic correspondences | | The children walk around the border of circles created on the floor with ropes while music is playing. When the music stops, the children have to jump in, experiencing the concepts of “border,” “inside,” and “outside”. |
| A.2 The hidden drawing  [3 tasks] | Making symbolic associations. Reconstructing the whole from single parts. | | The children are presented with a poster that hides an image, through a series of windows. Each window has a symbol on it (*e.g*., a star). The children extract cards from a bag and open the window that has printed on it the same symbol as that which they found on the card. When the windows are open, a detail of the hidden image is revealed. The children keep extracting cards and opening windows until they are able to guess what the image is. |
| A.3 Detective hunting for signs  [1 tasks] | Recognizing signs that change the graphic contextual meaning. | | Like a detective, the children have to look for signs that are the "same but different." The children are then exposed to 8 patterns including the elements of the human face (geometrical shapes representing face, eyes, eyebrows, and mouth) in a different order. The teacher asks if there are faces that are exactly the same, similar, opposite, or not plausible. |
| A.4 From-sign-to-sign  [3 tasks] | Identifying the differences and associations between similar images. | | A memory game with 16 pictures. Four categories are represented, within which there are two cards representing the same object (two cards representing a car and another two representing a similar car). |
| A.5 Lilli the explorer  [3 tasks] | Identifying the necessary signs to complete and attribute meaning to a drawing. | | First, the teacher reads the story of "Lilly the explorer." Meanwhile, the teacher shows the children pictures representing the main elements of the story. Then, the children are presented with other images and need to identify those that complete the pictures shown by the teacher. |
| **Coding** | | | |
| A.6 Lucia and Oreste the Giant  [3 tasks] | Recognizing signs that change the dimensions of an object. Representing objects of different dimensions. | | The children are told the story of "Lucia and Oreste the Giant," which helps them understand the concepts of "little" and "big." |
| A.7 Drawing the movement  [3 tasks] | Identifying the main elements to interpret an image. Using signs to transmit meanings. | | These activities help children use drawing to communicate. The children have to complete to drawings, one representing a bus stop, in which they have to add a little girl waiting for the bus, and another drawing representing a moving bus, in which they have to add a little girl running after the bus. |
| A.8 The signs of emotions  [3 tasks] | Attributing a semantic meaning to signs. Using the same signs to transmit different meanings on the basis of the context. | | The children are given several symbols and can glue them in order to represent different emotions (*e.g.,* joy, sadness). |
| A.9 Chasing the hatching  [3 tasks] | Experimenting with the correspondence between the parts and the whole. | | The teacher gives the children a poster representing a simple landscape, including shapes drawn with different hatching styles. The children have to trace over the shapes named by the teacher, paying attention to the hatching types. |
| A.10 Transform-action  [3 tasks] | Promoting divergent and creative thinking. | | The teacher shows the children some drawings representing shapes, and eth children have to reproduce them using, *e.g.,* sticks, ropes, and constructions. |
| **Targeting the orthographic sign** | | | |
| **Activities** | **Objective** | | **Example of task** |
| **Decoding** |  | |  |
| B.1 The writing box  [3 tasks] | Becoming familiar with common and uncommon writing tools. | | The teacher gives the children a box within which there are several objects for writing and drawing (*e.g.,* pencils, pens, paper, cardboard, chalks, and newspapers). The teacher asks the children some questions to stimulate discussion, for instance, what the name of such objects is, what the painter uses, or what their mom uses to make a shopping list. |
| B.2 The one who seeks finds, the one who thinks creates  [3 tasks] | Discriminating graphemes within complex patterns. | | The teacher presents the children with several complex shapes within which a letter is hidden (for instance, the letter "A" in the star of David) |
| B.3 Vocal circles  [3 tasks] | Recognizing vowels through visual-auditory associations. | | The teacher assigns each child a card with a vowel and places on the floor five circles, each corresponding to a vowel. At the teacher's indication, the children have to go inside the circle matching their assigned vowel. |
| B.4 Alphabetic parking lot  [3 tasks] | Recognizing letters and associating them with signs. Stimulating processes of phonological discrimination. | | Each child is assigned a "plate" with a vowel on it, and when the teacher (acting as a traffic policeman) raises the signaling disk with the children’s vowel on it, they have to go to the parking lot. |
| B.5 The fortune wheel  [3 tasks] | Reflecting on the correspondence between combinations of signs and meanings. | | The teacher gives each child a necklace with a letter on it. Then, the teacher places the children in a circle facing outwards. A child stays in the middle of the circle and is assigned a short word. The child has to touch his/her classmate and check what letter he/she has and keep him/her facing inwards only if it is a letter included in the word assigned. |
| **Coding** | | | |
| B.6 There is mail for you  [3 tasks] | Using writing for a purpose. Invented spelling. | | The teacher shows the children several materials associated with mail (*e.g.,* envelopes, letters, stamps, and postcards) and asks the children several questions, such as what are they for, have they ever been in a post office, what is the difference between a letter and a postcard? |
| B.7 The Lunaretti family  [3 tasks] | Stimulating phonological awareness through the recognition of foreign words. Experimenting with writing through the combination of signs. | | The teacher reads the story "The Lunaretti family," which includes some English words and short sentences that the children have to understand using the context. |
| B.8 Fish your classmate and read the word  [3 tasks] | Stimulating the process of phonological awareness and developing the ability to associate syllabic combinations with meaning. | | The children are given a necklace with a syllable on it. With the help of the teacher, each child in turn has to pick a classmate whose syllable on the neck forms a word with his/her own. |
| B.9 Pandolfa the frog  [3 tasks] | Recognizing and reproducing the same signs. Graphically representing the meaning corresponding to a combination of signs. | | The teacher reads to the children the story "Pandolfa the frog." Then, the teacher assigns the children some written words from the story associated with the image. The children are also assigned several cards with letters and have to reconstruct the word assigned by gluing the letters in the right order. |
| B.10 The “Us-you” dictation  [3 tasks] | Recognizing graphemes through visual-auditory correspondence. | | The children choose words and dictate them to the teacher, who has to write them on the blackboard. Sometimes, the teacher purposely commits a mistake, violating a characteristic of writing systems, such as repeating the same letter several times (*e.g*., writing goooooooose). The students have to stop and correct the teacher. |
| **Targeting the numeric sign** | | | |
| **Activities** | **Objective** | **Example of task** | |
| **Decoding** |  |  | |
| C.1 The numbers train  [3 tasks] | Stimulating phonological awareness through rhymes. Associating signs to quantities. | The teacher sings to the children a nursery rhyme about numbers and shows them a card with a number on it every time it is mentioned. | |
| C.2 Soap bubbles  [3 tasks] | Becoming familiar with Arabic numerals. | The teacher gives the children some shovels, each with a number from 1 to 9 on it. Then, a child starts blowing bubbles, and the teachers shows numbers: only the child with the shovel with the same number as that showed by the teacher can blow them up. | |
| C.3 The nine dwarves  [3 tasks] | Recognizing and pairing the same numerical signs. Ordering numbers from 1 to 9. | The children create some cone hats and put a number on them. Then, they are assigned a number and can make up a dwarf name. Subsequently, they have to find and put on the hat with their number on.it | |
| C.4 Fishing numbers  [3 tasks] | Ability to make multiple associations: the same numerals, numerals and colors, and numerals and movement. | The children play bingo with numbers. The teacher extracts numbers from 1 to 9, and the children have to color with the same color on their sheet all of the boxes reporting the extracted number. | |
| C.5 The exact time  [3 tasks] | Recognizing the numeral sign and associating it with an action. | The teacher creates a large clock reporting the numbers from 1 to 9. Then, each child constructs a little watch, reporting the same numbers, and moves the arrow to point to one of them. Then, they start singing; meanwhile, the teacher moves the arrow to point to a number. When the song finishes, all of the children whose watch signs the same number as the teacher's clock have to "fall down." | |
| **Coding** | | | |
| C.6 Let’s measure the temperature  [3 tasks] | Associating numerals with actions.  Recognizing and reproducing numerals. | The teacher shows a drawing of a thermometer and explains it. Then, he/she places the numbers in order, from 36 to 40. The children are given the same numbers and have to draw 36 in blue (cold) and 40 in red (hot). | |
| C.7 The number exhibition  [3 tasks] | Becoming familiar with numerals though creative activities. Associating numerals with quantities. | The teacher assigns a number from 1 to 9 to each child. The children have to find the classmates with the same number and decorate it, producing a painting about their number. | |
| C.8 Numbers in pieces  [3 tasks] | Reconstructing numerals and associating them with quantities. | The children are assigned a number printed in a large size. They have to color it. Then, the teacher cuts it in pieces, transforming it into a puzzle. The children then have to put all of the pieces back together. | |
| C.9 The numbers’ carnival  [3 tasks] | Recognizing and transforming numerals. Understanding the "ordinality" of numbers. | Each child is assigned a number and has to "dress it up." Then, the classmates have to identify which number hides under the costume. | |
| C.10 Numeric drawings  [3 tasks] | Discriminating the numeral from the background. Using the numeral to produce artistic productions. | The children have to complete a partially drawn picture using drawings of numbers. | |
